# Relative abundance and diversity of sharks and predatory fishes across Marine Protected Areas of the Tropical Eastern Pacific

**DOI:** 10.1371/journal.pone.0334164

**Published:** 2025-11-26

**Authors:** Simon J. McKinley, Sarah F. Hansen, Denisse Fierro-Arcos, Megan E. Cundy, Magdalena Mossbrucker, Gabriel M. S. Vianna, Jenifer Suarez-Moncada, Mauricio Hoyos-Padilla, Sandra Bessudo-Lion, Enric Sala, Pelayo Salinas-de-León

**Affiliations:** 1 Charles Darwin Foundation, Charles Darwin Research Station, Puerto Ayora, Galapagos Islands, Ecuador; 2 Galapagos National Park Directorate, Puerto Ayora, Galapagos Islands, Ecuador; 3 Pelagios-Kakunja A.C., La Paz, Baja California Sur, Mexico; 4 Fins Attached Marine Research and Conversation, Colorado Springs, Colarado, United States of America; 5 Fundación Malpelo y Otros Ecosistemas Marinos, Bogotá, Colombia,; 6 Pristine Seas, National Geographic Society, Washington DC, United States of America; 7 Guy Harvey Research Institute and Save Our Seas Foundation Shark Research Center, Nova Southeastern University, Dania Beach, Florida, United States of America; University of Messina, ITALY

## Abstract

Marine Protected Areas (MPAs) in the Tropical Eastern Pacific (TEP) support globally distinct reef fish populations, which exhibit differences between the remote oceanic islands and continental coast. While oceanic island MPAs typically support large abundances of sharks and large predatory teleost (bony) fishes, coastal MPAs show increasing signs of depletion. We deployed stereo-Baited Remote Underwater Video systems (stereo-BRUVs) to assess reef fish community structure across seven MPAs in the region. Oceanic island MPAs had considerably greater species richness and relative abundances than coastal MPAs across all trophic levels. Within the biogeographic subprovinces, fish assemblages were differentiated from each other corresponding to latitude, aligning with the established patterns and supporting finer scale bioregionalization within the TEP. Notably, oceanic MPAs supported some of the largest relative abundances (MaxN hr^-1^) of sharks on nearshore reefs reported globally. This is likely driven by the regional oceanographic processes enhancing productivity and trophic diversity and sustained by reduced anthropogenic disturbances associated with MPA remoteness and protection. Therefore, we highlight the critical role of MPAs in the TEP as refuges for sharks. However, we also found evidence of fishing pressure on predatory fishes within MPAs across the region. Coastal MPAs in Ecuador exhibit low fish abundances across all trophic levels, with large predators notably absent, indicative of ‘fishing down the food web’. Our results highlight the need for fishing impact assessments and improved conservation measures, such as strengthened enforcement, within MPAs to conserve globally significant marine biodiversity.

## Introduction

The Tropical Eastern Pacific (TEP), spanning the coastline from the Baja California Peninsula to northern Peru and encompassing many oceanic islands, represents a global biogeographic province characterized by distinct oceanographic processes and marine communities [1,2]. The TEP can be further subdivided into three subprovinces based on reef fish taxonomic and biomass patterns, with particularly pronounced differences in communities between the oceanic islands and continental coasts [2–4]. Oceanic islands exhibit higher levels of endemism in reef fish communities due to their isolation [2], while strong upwellings generated by ocean currents interacting with bathymetry around islands create localized high productivity hotspots that support dynamic food webs and sustain large populations of predatory fishes, including sharks [5–9]. In contrast, coastal reef fish communities in the TEP are influenced differently by oceanographic processes, including areas of weaker topographically induced upwellings and lower productivity, resulting in distinct species compositions [1,2]. Across the coastline, reef fish species richness gradually declines to the north and south of Costa Rica [2]. While predatory fish abundances are lower within coastal MPAs than the oceanic islands, some areas near the coast support notable predatory fish populations, such as Caño Island [10].

The TEP has a growing network of Marine Protected Areas (MPAs) aimed at conserving globally significant marine biodiversity and ecosystem functioning in the face of accelerating anthropogenic pressure [11–13]. Over the past decades, more than 77 MPAs have been designated in Mexico, Costa Rica, Panama, Colombia, and Ecuador, as well as oceanic islands within their Exclusive Economic Zones (EEZs). The MPAs vary in their level of protection, ranging from allowing extractive activities (e.g., Galera San Francisco Marine Reserve, Ecuador), to mixed-use management approaches (e.g., Galapagos Marine Reserve, Ecuador), to fully protected no-take zones (e.g., Malpelo Fauna and Flora Sanctuary, Colombia).

Reef fish assemblages across MPAs within the TEP are subject to distinct biogeographic, oceanographic and anthropogenic influences, which collectively shape and sustain the structure and function of these marine communities [1,2,4]. Due to the complex interplay of these factors, the effectiveness of these MPAs in conserving reef fish assemblages – particularly predatory fishes – remains inadequately assessed across the region’s distinct biogeographic subprovinces. Notably, anthropogenic pressure, including fishing, has impacted reef fish communities across MPAs in the subprovinces differently [4]. Coastal MPAs often experience high fishing pressure due to their proximity to human populations, while oceanic MPAs often benefit from remoteness [4,10,14,15]. But both experience challenges of effective enforcement [16,17], and illegal, unreported, and unregulated (IUU) fishing continues to impact fish populations, particularly predatory fishes [18–20].

Predatory fishes, including sharks and large teleost (bony) fishes, play crucial roles in maintaining ecosystem function and resilience, primarily by regulating food webs and recycling nutrients [ [21,22]]. However, these species are also among the most vulnerable due to their low resilience to fishing pressure [23,24]. In the TEP, predatory fish populations face significant fishing pressure from both targeted and incidental capture in fisheries [20,25–27]. Given the biogeographic, oceanographic and anthropogenic complexities, a comprehensive assessment of shark and predatory fish assemblages across MPAs of this diverse region may considerably assist conservation management. Fish census methods (e.g., underwater visual census and diver-operated video) are often used to sample reef fish but underestimate abundances of highly mobile and elusive species, such as sharks and predatory teleost fishes [28]. Alternatively, Baited Remote Underwater Video Systems (BRUVs) offer a standardized, non-extractive alternative that better represents predatory fishes in community analyses, including assessments of relative abundance and size structure [29]. Studies utilizing BRUVs across the globe have revealed variations in predatory fish populations across protection gradients, fishing pressures, including at regional scales and remote islands [30–33].

In the TEP, studies have documented reef fish relative abundance and size structure within individual MPAs or across fine-scale regions showing that MPAs generally support healthier marine populations than unprotected areas, even with limited enforcement [10,34–36]. Yet coastal MPAs in some regions, such as Ecuador, remain significantly underrepresented in research. This knowledge gap raises concerns about the overall health of reef fish communities and the population status of predatory fishes along much of the TEP coastline that experiences heavy fishing pressure [4]. Moreover, despite TEP’s recognition as a global shark hotspot [7,37,38], a standardized assessment of shark relative abundances and size structures across MPAs is yet to be conducted [33,39].

Therefore, we conducted the first assessment of sharks and predatory teleost fish communities using BRUVS across MPAs in the TEP. Specifically, our study aimed to compare community composition, abundances, and size structure between coastal and oceanic MPAs. We hypothesized that sharks and predatory teleost fish assemblages:

(1) would differ between coastal and oceanic MPAs, reflecting broad-scale biogeographical and oceanographic differences;
(2) would vary according to local environmental conditions and level of protection among MPAs within each biogeographic subprovince;
(3) composition and length frequency distributions would potentially indicate fishing pressure on sharks and commercially valuable teleost fishes.

## Materials and methods

### Ethics statement

This research was conducted under permits from the Galapagos National Park Directorate for the Galapagos Marine Reserve (PC-28–16 & PC-27–17); the Ecuadorian Minister of Environment for Machalilla National Park (006–2019-DP-DPAM-MAE) and Galera San Francisco Marine Reserve (007–2019-IC-FLO-FAU-DPE-MAE); the Haut commissariat de la République en Polynésie Francaise French (HC167CABBSIRIMG) and Direction générale de la mondialisation, de la culture, de l’enseignement et du development international (2016_177320/DGM/DCERR/ESR) for Clipperton Atoll; the Direction of the Revillagigedo Archipelago Biosphere Reserve for Revillagigedo (F00.DRPBCPN.DIR.RBAR.-032/2016); the Direction of the Natural National Parks of Colombia and Malpelo Foundation (Convenio de Asociación 003/2013–2018) for the Malpelo Flora and Fauna Sanctuary; the National System of Conservation Areas (SINAC-ACOSA-INV-010–19 & SINAC-ACOSA-PI-PC-025–19) for Isla del Caño Biological Reserve.

Fish were recorded in their natural habitat by video cameras using a non-invasive technique without capture, handling or physical disturbance. No animal ethics approval was required for such observational research. Bait used to attract fish was sourced locally using permitted fishing techniques.

### Fish community data

#### Study sites.

**Fig 1:**
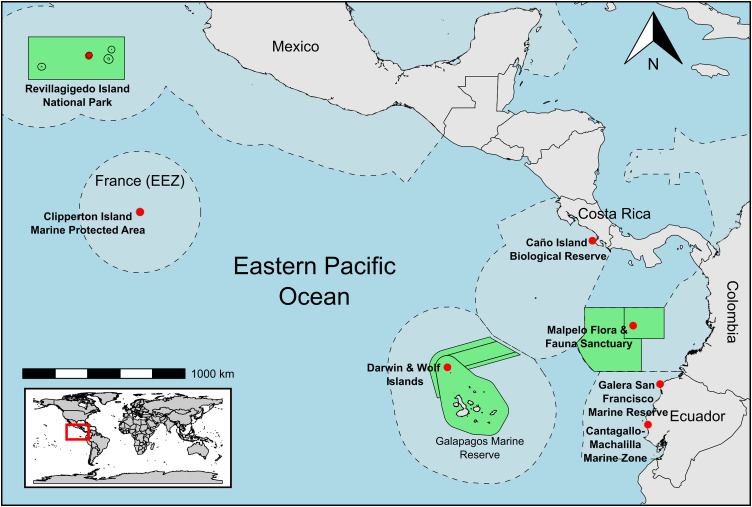
The seven Marine Protected Areas (MPAs) sampled by stereo-BRUVs within the Tropical Eastern Pacific (TEP). Base map made with Natural Earth data (Free vector and raster map data @ naturalearthdata.com); MPA boundaries from the World Database of Protected Areas (IUCN and UNEP-WCMC (2025), The World Database on Protected Areas (WDPA) [On-line], August 2025, Cambridge, UK: UNEP-WCMC. Available at: www.protectedplanet.net.WDPA Updates).

This study assessed reef fish assemblages within MPAs across the TEP, a biogeographically distinct marine region globally (Fig 1). The TEP extends from the Baja California Peninsula (Mexico) in the north (~25°N) to the northern coast of Peru (~4°S), encompassing coastal areas and oceanic islands out to approximately 120°W, which comprise distinct coastal and oceanic subprovinces [2,40].

Our study sampled reef fish assemblages within seven MPAs across the coastal and oceanic subprovinces of the TEP (Fig 1 and Table 1). The Galera San Francisco Marine Reserve (herein, Galera) and Cantagallo-Machalilla Marine Zone (herein, Machalilla) are located along Ecuador’s continental coast, and the Caño Island Biological Reserve (herein, Caño) is located within 15 kilometres of Costa Rica’s coast so were classified as coastal MPAs. The other four sites in Revillagigedo Archipelago National Park (herein, Revillagigedo), Clipperton Island Marine Protected Area (herein, Clipperton), Malpelo Fauna and Flora Sanctuary (herein, Malpelo), and Darwin and Wolf Marine Sanctuary in the Galapagos Marine Reserve (herein, Galapagos), are located at oceanic islands with a minimum distance of 380 km from the coast and were classified as oceanic MPAs.

**Table 1:** Summary of the seven Marine Protected Areas (MPAs) sampled using stereo-BRUVs in the oceanic island and coastal biogeographic subprovinces of the Tropical Eastern Pacific (TEP). Multiple use areas are not fully protected including designated zones for science, tourism or fishing.

	Marine Protected Area (Country)	Level of protection	Area Protected (Year Established/Expanded)	BRUVS	Month Year sampled
**Oceanic island**	Revillagigedo Archipelago National Park (Mexico)	Multiple use area	4.4 km^2^ (1994)	10	April 2016
		No-take	148,800 km^2^ (2017)		
	Clipperton Island Marine Protected Area (France)	No-take	1,811 km^2^ (2016)	21	March 2016
	Malpelo Fauna & Flora Sanctuary (Colombia)	No-take	651 km^2^ (1995) 8,575 km^2^ (2005) 27,096km^2^ (2017)	19	September 2015 & April 2018
	Darwin & Wolf Islands, Galapagos Marine Reserve (Ecuador)	Multiple use area (0.96% no-take)	133,000 km^2^ (1998)	15	May 2016 & March 2017
		No-take	40,000 km^2^ (2016)		
**Coastal**	Caño Island Biological Reserve (Costa Rica)	No-take	55.3 km^2^ (1978)	10	March 2019
	Galera San Francisco Marine Reserve (Ecuador)	Multiple use area	546 km^2^ (2009)	11	April 2019
	Cantagallo-Machalilla Marine Zone (Ecuador)	Multiple use area	144.3 km^2^ (1979)	25	August 2019
		Multiple use area	1,423 km^2^ (2015)		

The coastal MPAs were all located within the equatorial climate zone from Costa Rica to Ecuador, a region that experiences strong geographic variation in marine environmental conditions driven by current systems and ENSO events [41]. Reef fish communities include tropical and sub-tropical species, with species composition and richness gradually declining to the north of Costa Rica and south of Panama [2]. Caño marine habitats are composed of rocky and coral reefs, with water temperatures ranging from 26–30°C throughout the year [42]. The Ecuadorian MPAs feature less coral and more rocky reef substrate than Caño, and water temperatures are generally cooler [43]. Due to their proximity to large human populations and fishing ports, and less management enforcement than the oceanic MPAs, these coastal MPAs are likely subject to more fishing pressure.

Oceanic MPA’s ranged in latitude from 0.6°S to 18.84°N with distances of between 380 and 1075 km from the continental coastline [2]. Each oceanic MPA has distinct marine conditions characterized by its geographic position and influences of regional ocean currents [1,44]. Reef habitats consist of insular shelves, with Clipperton having more extensive coral reef development than Revillagigedo, Malpelo and Galapagos where rocky substrate is more prominent [45,46]. The remoteness of these MPAs is assumed to reduce fishing pressure on reef fish assemblages when compared to coastal MPAs [4]. Notably, sampling occurred prior to the expansion of protected areas at Clipperton (2016), Revillagigedo (2017), and implementation of the no-take Darwin and Wolf Marine Sanctuary at Galapagos (2016).

### Sampling design

Reef fish assemblages were opportunistically sampled at the seven MPA’s between September 2015 and August 2019 using stereo Baited Remote Underwater Stereo-Video (stereo-BRUVs) (Table 1). The soak time and minimum number of BRUV deployments per site followed recommendations for sampling shark and fish assemblages in the Galapagos, aiming to obtain adequate spatial coverage while maintaining minimum site-level replication (n = 4 deployments per site), including at small islands with limited available reef habitat [34,47]. Sampling always used a spatially stratified design with random replicate samples within each of the MPAs. Stereo-BRUVS were deployed at approximately 20–25 meters depth and with a minimum distance of 500 m between replicate deployments to avoid overlapping of bait plumes and minimize the risk of the same individual appearing in videos of successive deployments [48,49]. Deployments had at least 100 minutes of bottom time to provide 90 minutes of video for analysis as the first 5 and last 5 minutes of footage were discarded to mitigate the disturbance caused by the boat to the fish community sampled during deployment [34]. All deployments were completed during daytime hours between 7:00 and 14:00.

### Stereo-BRUVs

Stereo-BRUVs are a non-intrusive tool to sample reef fish assemblages [50]. They have been shown to observe larger abundances of predatory species than diver-operated videos or underwater visual census without impacting observations of lower trophic level species [28,48]. Each of our stereo-BRUVs consisted of a triangular stainless-steel frame and two GoPro Hero 4 cameras in waterproof housings mounted to the base bar 70 cm apart, angled inwards at 7**°** degrees and orientated horizontally to the seafloor. GoPro’s recorded video footage at medium field of view, 1080 pixels and 60 frames per second. A bait canister holding 800 grams of chopped yellow fin tuna (*Thunnus albacares*) with the skin was positioned in the cameras field of view attached at the end of a 1.3-meter PVC pipe. The frame was attached to a buoy at the surface and anchored a 20 kg weight on the seabed to keep the stereo-video system floating approximately 1 meter above the substrate, a design shown to reduce entanglements in structurally complex and exposed habitats, and with large animals [34,47]. This was particularly important for the high current areas in Galapagos and at the coral reef surrounding Clipperton Islands.

### Video analysis

Stereo-BRUVs were calibrated before each fieldtrip using SeaGIS CAL software (https://www.seagis.com.au/bundle.html) following standardised procedures [51]. For each stereo-BRUV deployment, 90 minutes of video footage was analyzed using EventMeasure software (https://www.seagis.com.au/event.html). All cartilaginous and teleost (bony) fishes were identified to the lowest possible taxonomic level and the relative abundance of each species in a video was determined as the maximum number of individuals of taxa visible in one still frame (MaxN). Fish that could not be confidently identified to species were identified to genus or family. MaxN is used to avoid counting an individual more than once during the video and is therefore a conservative estimate of species relative abundance [50]. To standardize sampling effort, the MaxN of species in each deployment was divided by the time used for video analysis and expressed as MaxN hr^-1^. This was necessary because three systems stopped filming before reaching 90 minutes, while also allowing for comparisons of shark’s abundances globally (Table 5). Fork lengths of teleost and shark species and disc width of ray species were measured in stereo-videos at the time the species MaxN was counted. Measurements with a root mean square (RMS) value greater than 20 mm were considered imprecise and excluded.

### Trophic groups

Species were categorized into five trophic groups using diet and feeding information from FishBase [58]. Sharks and high-order teleost fishes were considered as two distinct groups that predominantly feed on large prey fishes and invertebrates and focal species of the study due to being fished in the region or being of conservation concern [59–61]. Sharks were separated because they generally grow larger and have different life-histories compared to reef-associated teleost fishes [62]. The high-order group consisted of large predatory teleost fishes (i.e., generally growing larger than 80 cm), and included benthopelagic carangids, lutjanids, and serranids. Meso-predators feed on a wide range of prey species, generally smaller than those consumed by high-order species. This group therefore included benthic and demersal predators, as well as smaller species from high-order predator families. Planktivores predominantly feed on organisms suspended in the water column. This group included filter-feeding elasmobranchs, benthopelagic schooling species (e.g., some balistids and *Cephalopholis colonus*), and certain pomacentrids. Herbivores predominantly feed on macroalgae on the substrate. This group generally consisted of demersal species that roam reefs (e.g., some acanthurids, kyphosids, and scarids) or exhibit site fidelity (e.g., some pomacanthids, chaetodonids, and pomacentrids).

### Statistical analysis

#### Univariate statistics.

To describe reef fish communities, species richness hr^-1^ and MaxN hr^-1^ were calculated for each trophic group in each deployment. Bar and boxplots were then constructed using the *ggplot2* package in R comparing these means of these metrics across MPA’s [63]. Both metrics were recalculated for the whole community in each deployment, Euclidean distances calculated between deployments, and differences between fish communities tested using a two-way nested Permutational Multivariate Analysis of Variances (PERMANOVA, α = 0.05) with the factor’s biogeographic ‘subprovince’ (fixed, two levels) and MPA (random, 7 levels) nested within subprovince. PERMANOVA procedures were performed using PRIMER v7 with the PERMANOVA+ package [64,65].

#### Multivariate patterns.

The same two-way nested factor design was used to assess multivariate patterns in the whole fish community data and datasets for each trophic group. Fish species relative abundances (MaxN hr^-1^) in each dataset were square-root transformed to reduce the influence of highly abundant species on dissimilarity calculations and distances between deployments calculated using the Bray-Curtis index of dissimilarity with a dummy variable (+1) added to each deployment. The zero-adjusted Bray-Curtis index was used as it allows for distances to be calculated in assemblage data that naturally has many 0’s while avoiding undefined values when deployments had no species in common [66,67]. Assessments of the terms in the full PERMANOVA models were conducted using Type III sum of squares using 9999 permutations under a reduced model [68]. To address potential confounding effects of biogeographic variation on MPA comparisons, we conducted separate PERMANOVA analyses within each biogeographic subprovince to further assess differences in fish assemblages among MPAs. Assessments of terms in the full PERMANOVA models and pairwise tests were conducted using Type III sum of squares with 9999 permutations under unrestricted permutations of raw data and Monte Carlo bootstrapping for low sample sizes [69].

The whole fish community data was further assessed using Canonical Analysis of Principal Coordinates (CAP, α = 0.05) with leave-one-out allocation to distinguish hypothesised groups (MPAs) in multivariate space. Species contributing to the observed differences between MPAs were considered to have strong Pearson correlations when canonical axes were above |r| > 0.7. Of these species, focal species were plotted on the CAP plot. All multivariate procedures were performed using PRIMER v7 with the PERMANOVA+ package [64,65].

#### Fork length frequency distributions.

The length frequency distributions of focal species were also plotted using the *ggplot2* package in R [63]. These species included Galapagos sharks (*Carcharhinus galapagensis*), scalloped hammerhead sharks (*Sphyrna lewini*), Dermatolepis *dermatolepis*, *Mycteroperca olfax*, *Caranx lugubris, C. melampygus, and C. sexfasciatus*. The proportion of immature individuals of species with ten or more measurements in an MPA were described using the smallest published length of sexual maturity estimates [70–75]. No reliable maturity estimate was available for *D. dermatolepis*.

## Results

The 111 benthic BRUVs deployed detected a total of 18771 individual fishes belonging to 52 families and 181 species. Overall, 8 species were sharks, 9 were classified as high-orders, 107 as meso-predators, 17 as planktivores and 40 as herbivores.

### Differences in fish assemblages

#### Univariate statistics.

**Fig 2:**
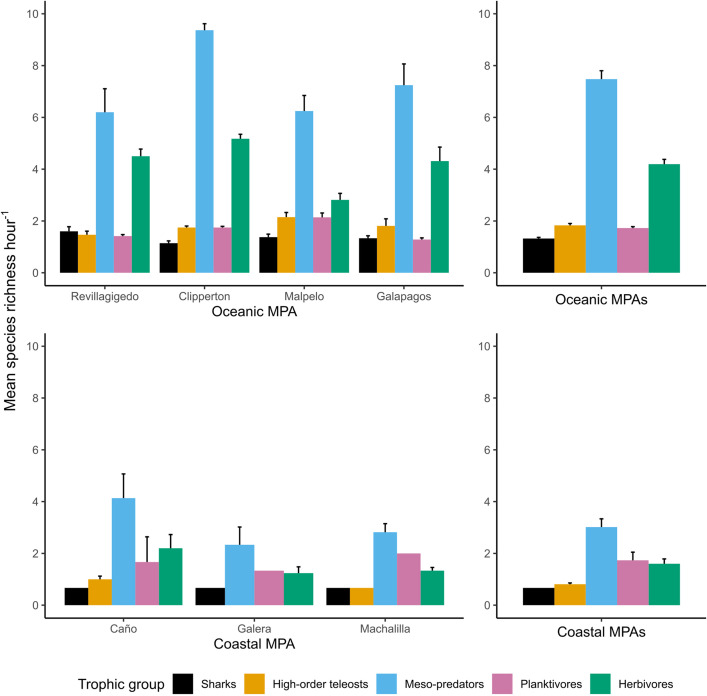
Mean species richness hr^-1^ of reef fish trophic groups sampled by stereo-BRUVs in seven TEP MPAs.

The species richness hr^-1^ differed significantly between the biogeographic subprovinces and MPAs within them (Table 2). MPAs in the oceanic subprovince had a higher species richness hr^-1^, on average, than the coastal subprovince (Fig 2). There was also higher species richness hr^-1^, on average, in each trophic group in the oceanic subprovince than in the coastal, where there were only two shark and three high-order teleost species detected (Fig 2). Within the oceanic subprovince, mean species richness hr^-1^ in each trophic group was generally similar across MPAs, albeit with some variation in the meso-predator group across MPAs. Within the coastal subprovince, species richness hr^-1^ was slightly higher, on average, in Caño. Notably, no high-order teleost species were detected in Galera.

**Table 2:** Two-factor nested PERMANOVA for differences in reef fish species richness hr^-1^ and relative abundance (mean MaxN hr^-1^) across seven MPAs within the TEP. Biogeographic subprovince is a fixed factor and MPA is a random factor nested within subprovince. Both metrics dissimilarity between stereo-BRUV deployments was calculated using Euclidean distances. Bold emphasise significant differences at α = 0.05.

Metric	Factor	Degrees of freedom	Mean squares	Pseudo-F statistic	Permutational P-value
Species richness hr ⁻ ¹	Subprovince	1	2892.8	35.407	**0.018**
	MPA(Subprovince)	5	87.414	4.3623	**0.002**
Mean MaxN hr ⁻ ¹	Subprovince	1	260.19	2.351	0.108 0.151
	MPA(Subprovince)	5	119.57	8.109	**<0.001**

**Fig 3:**
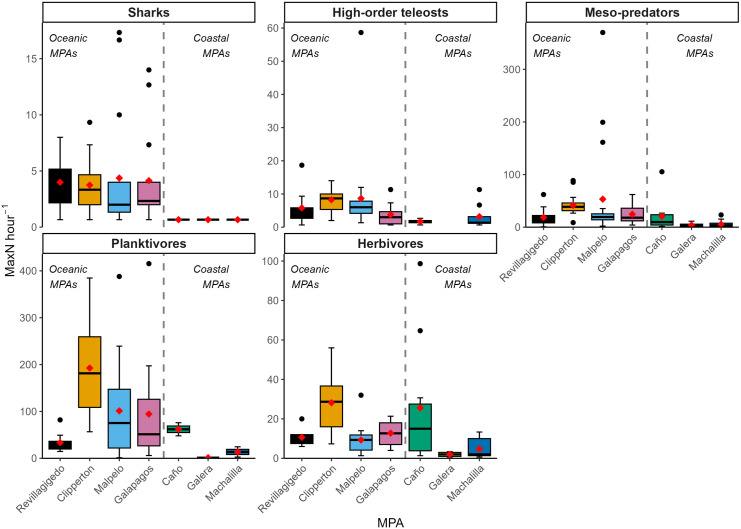
Relative abundances (MaxN hr^-1^) of reef fish trophic groups sampled by stereo-BRUVS across seven TEP MPAs. Boxplots display means (red circle), medians (black line), upper and lower quantiles (boxes), minimum and maximum + − 1.5*interquartile range (whiskers) and outliers (black dots).

On average, relative abundances of all trophic groups were larger in MPAs in the oceanic than in the coastal subprovince (Fig 3). A statistically significant difference in mean MaxN hr^-1^ was detected between the MPA’s within the subprovinces. While MaxN hr^-1^ was higher, on average, in MPA’s of the oceanic subprovince, there was no statistical difference detected in mean MaxN hr^-1^ between subprovinces (Table 2). Within the oceanic subprovince, relative abundances of all trophic groups were, on average, largest in Clipperton. Within the coastal subprovince, relative abundances of planktivores and herbivores were, on average, larger in Caño. Shark relative abundances (mean MaxN hr^-1^) in the TEP oceanic MPAs were some of the largest reported globally when compared to other studies in reef habitats using BRUVs at comparable depths (Table 5).

**Table 4:** Success of the leave-one-out allocation of sites to the seven TEP MPAs. Total misclassification error was 11.7%.

Subprovince	Original MPA	Classified MPA							
		Revillagigedo	Clipperton	Malpelo	Galapagos	Cano	Galera	Machalilla	Correct (%)
**Oceanic island**	Revillagigedo	10	0	0	0	0	0	0	100
	Clipperton	0	21	0	0	0	0	0	100
	Malpelo	0	0	16	2	0	0	1	84.211
	Galapagos	1	0	1	13	0	0	0	86.667
**Coastal**	Cano	0	0	0	0	9	0	1	90
	Galera	0	0	0	0	0	8	3	72.727
	Machalilla	0	0	0	1	0	3	21	84

### Multivariate patterns

Results of the multivariate PERMANOVA for relative abundances were similar for most tests of the fish community and trophic group datasets (Table 3). The whole community, shark, high-order teleost, meso-predator and planktivore fish assemblages differed significantly among and within subprovinces. Herbivore fish assemblages differed significantly between MPAs but not subprovinces. Multivariate PERMANOVA and pairwise tests within each subprovince found fish communities differed significantly among all oceanic and among all coastal MPAs (S1 Table).

**Table 3:** Two-factor nested PERMANOVA for differences in whole reef fish community and five trophic group multivariate composition across seven MPAs in the TEP. Subprovince is a fixed factor and MPA is a random factor nested within subprovince. Relative abundances (MaxN hrs^-1^) were square-root transformed and dissimilarity between stereo-BRUV deployments calculated using Bray-Curtis with a dummy variable (+1). Bold emphasise significant differences at α = 0.05.

Dataset	Factor	Degrees of freedom	Mean Squares	Pseudo-F statistic	Permutational P-value
Whole community	Subprovince	1	68,751	4.837	**0.001**
	MPA(Subprovince)	5	15,353	7.954	**<0.001**
Sharks	Subprovince	1	35,878	6.732	**<0.001**
	MPA(Subprovince)	5	5,781.70	12.783	**<0.001**
High-order teleosts	Subprovince	1	40,681	4.074	**0.028**
	MPA(Subprovince)	5	54,301	16.798	**<0.001**
Meso-predators	Subprovince	1	60,348	5.175	**0.011**
	MPA(Subprovince)	5	12,572	6.812	**<0.001**
Planktivores	Subprovince	1	90,718	15.739	**0.013**
	MPA(Subprovince)	5	6,219.60	7.354	**<0.001**
Herbivores	Subprovince	1	41,738	2.279	0.052
	MPA(Subprovince)	5	19,903	16.449	**<0.001**

**Fig 4:**
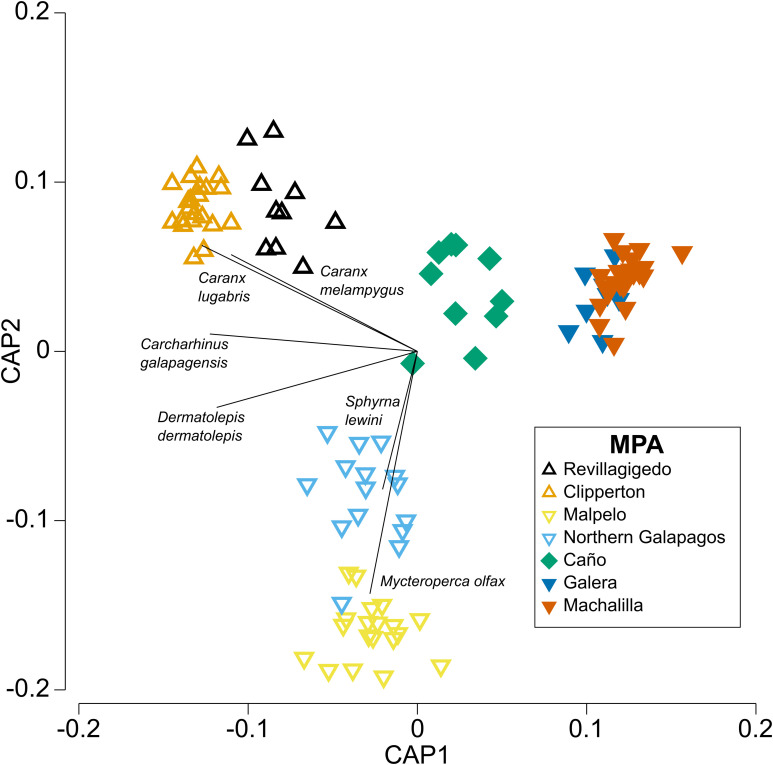
Canonical Analysis of Principal Coordinates (CAP) ordination of reef fish multivariate composition across seven TEP MPAs.

The CAP plot for the whole fish community showed four groups (Fig 4). The oceanic island MPAs were separated into two groups, one included Revillagigedo and Clipperton and the other, Galapagos and Malpelo. The coastal MPAs were also separated into two groups, one included the Ecuadorian MPAs of Galera and Machalilla, and the other Caño. All Revillagigedo and Clipperton deployments were correctly reclassified by the CAP procedure (Table 4). Misclassifications included two Malpelo deployments assigned to Galapagos, and a Galapagos deployment assigned to Malpelo and Revillagigedo, respectively. Among coastal MPAs, one Caño deployment was misclassified as Machalilla, three Machalilla deployment were misclassified as Galera and one as Galapagos, and three Galera deployment were misclassified as Machalilla.

Eighteen species were strongly correlated (>0.7) with the first or second canonical axes (S1 Table), including focal species (Fig 4). *Myteroperca olfax* and *Sphyrna lewini* were positively correlated to the second axis directed towards where Malpelo and Galapagos were grouped. *Caranx lugubris* and *C. melampygus* were negatively correlated to the first canonical axis directed towards where Revillagigedo and Clipperton were grouped. *Carcharhinus galapagensis* and *Dermatolepis dermatolepis* were negatively correlated to the first canonical axis and away from the coastal MPAs.

### Focal species length frequency distributions

**Fig 5:**
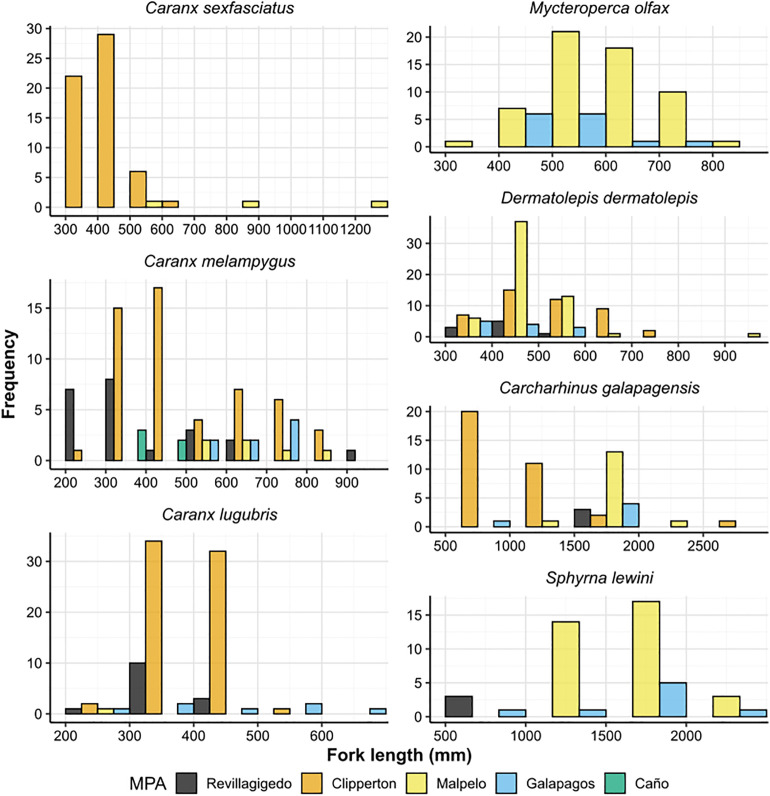
Length frequencies of focal predatory fish species sampled using stereo-BRUVs in TEP MPAs.

Focal species were mostly measured at oceanic island MPAs, while measurements were not recorded for these species at coastal MPAs due to low relative abundances or absences from samples, except for 5 *Caranx melampygus* at Caño (Fig 5). Fork length-frequency distributions patterns varied among species and MPAs (Fig 5). Focal species with ten or more measurements and more than 50% of individuals measuring below estimated sizes of sexual maturity included *Mycteroperca olfax at* Malpelo (69%) and Galapagos (93%), *Caranx sexfasciatus* at Clipperton (81%), *C. melampygus* (59%) and *C. lugabris* at Revillagigedo (57%) and *Carcharhinus galapagensis* (97.1%) at Clipperton (59,110–114).

## Discussion

Our study found significant differences in shark and predatory fish assemblages between oceanic island and coastal MPAs. Oceanic islands MPAs had higher species richness and larger relative abundances across all trophic levels, including some of the largest shark abundances reported globally (Table 5), highlighting the region’s distinct oceanographic and ecological processes that support larger reef fish populations. Similar to the results of Edgar et al. [11], we also argue that there is evidence of fishing pressure in MPAs of the TEP. Within coastal MPAs in Ecuador, sharks and large predatory fishes were mostly absent despite using a methodology designed to effectively survey them, while fish abundances across trophic levels were low indicating ‘fishing down the food web’ [4,76].

### Shark abundances in oceanic MPAs

We report some of the largest relative abundances of sharks at the oceanic MPAs in the TEP compared to reports at comparable depths from other regions globally (Table 5; [39,52]). Some of these studies focused on coastal reefs or areas nearer to human populations, where shark populations are more likely to be depleted by overfishing and other anthropogenic disturbances [77,78]. Other studies examined isolated island reefs that are less impacted by direct human activity [31,32,79,80] and reported lower shark abundances than we observed. We suggest that the combination of remoteness, reduced direct human impact, and the unique biophysical setting of oceanic islands in the TEP allows sharks to exhibit such large abundances.

**Table 5:** Comparison of some of the largest reported shark relative abundances (mean MaxN hr^-1^) sampled in reef habitats at comparable depths by BRUVs globally. Values of the most abundant species in each study and abundant species in our study of the TEP are presented (i.e., *Sphyrna lewini* and *Carcharhinus albimarginatus*). Additional values are reported in Table 6 by [52].

Region	Location (Year)	Deployments (mins)	Mean shark MaxN hr^-1^ ± SD	Mean species MaxN hr^-1^ ± SD
Tropical Eastern Pacific	Revillagigedo Archipelago, Mexico (2016) ^a^	10 (90)	4 ± 2.2	*Triaenodon obesus* 2.17 ± 1.11, *Carcharhinus albimarginatus* 1.8 ± 1.14, *Carcharhinus galapagensis* 1.07 ± 0.6
	Clipperton Island, France (2016) ^a^	21 (90)	3.75 ± 2.27	*Carcharhinus galapagensis* 2.98 ± 2.15, *Carcharhinus albimarginatus* 1.27 ± 0.86
	Malpelo Island, Colombia (2015, 2018) ^a^	19 (90)	4.38 ± 5.43	*Sphyrna lewini* 4.37 ± 6.62, *Carcharhinus galapagensis* 1.53 ± 1.48, *Triaenodon obesus* 1.21 ± 1.19
	Darwin & Wolf Islands, Galapagos Marine Reserve, Ecuador (2016, 2017) ^a^ This study	15 (90)	4.14 ± 4.25	*Sphyrna lewini* 3.67 ± 3.91, *Carcharhinus galapagensis* 0.94 ± 0.6
	Costa Rica (2016–2019) [10]	430 (103^b^)	NA	*Sphyrna lewini* 7.4 ± 11.1, *Triaenodon obesus* 3.7 ± 3.5, *Carcharhinus albimarginatus* 1.7 ± 1.6, *Carcharhinus galapagensis* 1.6 ± 1
	Islas Murcielago Archipelago, Costa Rica (2017–2019) [36]	67 (90)	1.5 ± 0.2 (Carcharhinidae spp. only)	*Carcharhinus falciformis* 1 ± 0, *Carcharhinus leucus* 1.3 ± 0.5, *Carcharhinus limbatus* 1 ± 0, *Galeocerdo cuvier* 1 ± 0, *Triaenodon obesus* 2 ± 1.9
	Bocos del Toro Archipelago, Panama (2016–2019) [53]	149 (65)	NA	*Ginglymostoma cirratum* 0.4, *Carcharhinus limbatus* 0.01, *Carcharhinus perezi* 0.01, *Sphyrna lewini* 0.0067
Indo-Pacific	Tetiaroa Atoll, French Polynesia (2016) [54]	42 (60)	NA	*Carcharhinus melanopterus* 1.71 ± 1.13, *Negaprion acutidens* 0.36 ± 0.62
	French Polynesia (2016–2017) [55]	2015 (60)	2.45 ± 2.27	*Carcharhinus melanopterus* 1.32 ± 1.25, *Carcharhinus amblyrhynchos* 0.74 ± 1.29, *Triaenodon obesus* 0.2 ± 0.45, *Sphyrna lewini* 0.004 ± 0.07
	^2^TRNP, Philippines (2015–2016) [56]	26 (60)	1.96 ± 2.05 ^c^	*Carcharhinus amblyrhynchos* 1.31 ± 2.94 ^c^, *Trianodon obesus* 1.04 ± 0.45 ^c^, *Sphyrna lewini* 0.04 ± 0.2 ^c^
		46 (60)		*Carcharhinus amblyrhynchos* 0.52 ± 1.01 ^c^, *Trianodon obesus* 0.74 ± 0.44 ^c^
	Middleton Reef, Australia (2020) [57]	71 (60)	NA	*Carcharhinus galapagensis 2.54, Galeocerdo cuvier 0.11*
Indian Ocean	BIOT, (2012) [32]	138 (60)	1.97 ± 0.35	*Carcharhinus amblyrhynchos* 1.33 ± 0.29, *Trianodon obesus* 0.17 ± 0.09, *Carcharhinus albimarginatus* 0.17 ± 0.09, *Sphyrna lewini, Sphyrna mokarran & Galeocerdo cuvier* <0.07

*Note*: TRNP = Tubbataha Reefs Natural Park, BIOT = British Indian Ocean Territory Marine Reserve. ᵃ This study, ^b^ = mean soak time, ^c^ = cumulative (cMaxN) from shallow reef (<15 meters) surveys.

We also found variations in shark assemblages among the oceanic MPAs. Notably, the silvertip shark (*Carcharhinus albimarginatus*) was exclusively observed at the northern MPAs, while the scalloped hammerhead shark (*Sphyrna lewini*) exhibited substantially larger relative abundances at the equatorial MPAs. While having a broad distribution across the Pacific and Indian Oceans [81], the silvertip shark is more commonly recorded in areas north of Galapagos and Malpelo, including our study sites and Cocos Island National Park [82]. This suggests a limited latitudinal range of the species within the TEP. The large abundance of scalloped hammerhead sharks at the equatorial MPAs align with previous studies reporting large aggregations at these locations [7,10,83]. These areas, characterised by strong upwelling and productive waters, as well as the presence of deep seamounts and coastal drop-offs, provide ideal conditions for schooling behaviour, feeding, cleaning and mating, likely explaining the observed large abundances at specific times during the year [84–87].

### Differences between biogeographic subprovinces

Fish assemblages in oceanic island MPAs differed significantly from those in coastal MPAs, with oceanic islands having higher relative species richness across most trophic groups. This aligns with the island biogeography theory, which predicts that isolation promotes speciation over evolutionary timescales, resulting in more distinct species and higher species richness [88]. This increased endemism distinguishes oceanic island MPAs from the coastal MPAs, not only in species diversity, but also in taxonomic composition [2,41,89].

Oceanic island MPAs also hosted larger relative abundances across most trophic groups than coastal MPAs. This likely results from a combination of oceanographic processes and anthropogenic impacts. The oceanic islands often experience enhanced productivity [1,90]. This supports dynamic food webs at nearshore reef habitats, from lower trophic level abundances of planktivorous and herbivorous fish, to higher trophic levels of meso-predator and high-order teleost’s, and sharks [91–93]. This productivity creates marine biodiversity hotspots where migratory species aggregate alongside resident reef species, resulting in increased abundances across trophic levels.

But the most striking difference between oceanic and coastal MPAs was the near absence of sharks and large predatory teleost fish in coastal MPAs, which is particularly noteworthy considering BRUVs typically attract predatory fishes [28,29]. Although robust scientific sampling of predatory fish populations along the coastline remains limited, our observations align with anecdotal evidence and previous studies documenting the low abundance of large predatory fishes in coastal MPAs in Ecuador [4,94–96] and within neighbouring countries [97]. Non-selective fishing gears (e.g., longlines, trawls, and gillnets) have been used near these coastlines for decades [95,98], which can rapidly deplete species populations, especially when unmanaged or used in the same area [99]. Additionally, the prevalence of IUU fishing has likely led to depletions [94,100]. Our findings, in conjunction with the aforementioned studies, support the hypothesis that fishing pressure, exacerbated by ineffective enforcement, is likely a driver of the observed low predatory fish abundances [4]. Future studies comparing fish communities in protected and unprotected areas at varying distances from human populations, incorporating quantitative fishing pressure indices (e.g., vessel tracking data and landing statistics), would provide stronger inferences about the relative contributions of biogeography versus protection in structuring reef fish assemblages.

The low fish abundances across trophic levels further supports the fishing of predatory fishes hypothesis, and the argument of “fishing down the food web” in coastal Ecuadorian MPAs [4]. While we found lower trophic level fish abundances in Caño that do not demonstrate fishing down effects, the notable lack of large predatory fish may suggest fishing impacts inside or outside the MPA of some wide-ranging species [10,101].

In several oceanic MPAs, the length frequency distribution of commercially valuable species of carangids and serranids peaked below estimated sizes of sexual maturity. This could reflect habitat preferences, as larger individuals may inhabit deeper waters than our BRUV deployments depths of 20–25 meters [102,103], or indicate fishing pressure effects [104]. Documented fishing impacts exist for some targeted species in the region, such as declines in *M. olfax* abundance and size in Galapagos [105]. However, the effects of fishing remain largely unknown for many commercially valuable species across MPAs in the TEP due to limited data on catch and fishing effort, as well as trends of population abundance. Prompt, comprehensive assessments of the population health of fished species within MPAs across the region, including sampling across depth strata, are needed to inform effective conservation and fisheries management strategies.

### Differences within biogeographic subprovinces

Our grouping of MPAs within the oceanic island and coastal subprovinces based on latitude aligns with established biogeographic patterns for reef fish in the TEP [2,3,41]. While the biogeographic patterns are well-documented, some observations are worth noting. Within the oceanic subprovince, the northern islands (Revillagigedo and Clipperton) were distinct from the islands closer to the equator (Galapagos and Malpelo). As trophic group relative species richness and abundances were generally similar across these locations, these findings support finer scale bioregionalization within the TEP based on taxonomic composition rather than community structure. Clipperton exhibited the largest overall fish abundances across trophic groups, possibly reflecting its the greater alive coral coverage reef relative to rocky reef, which can support higher fish densities due to more structural complexity [8,43,106,107]. Conversely, while Revillagigedo and Clipperton share similarities in taxonomic composition at a broad level, Clipperton hosts higher endemism [8], and their marine community structure may also differ due to habitat variations.

Within the coastal subprovince, Caño was distinct from the coastal Ecuadorian MPAs, exhibiting larger abundances of planktivorous and herbivorous fishes. This may reflect Caño’s greater extent of coral reef relative to rocky reef, which typically host higher reef fish densities [108,109]. We also suggest it’s slight offshore position, bathymetry and, proximity to the Costa Rica Dome (CRD) supports reef fish assemblages with some of the characteristics of the islands further offshore [1,110]. For example, the planktivorous schooling fish, *Cephalopholis colonus,* was prevalent, a species highly abundant at offshore islands [7,8]. This reinforces that oceanographic processes are key drivers of reef fish assemblages within the TEP. Future biogeographic studies should consider local oceanographic processes that are key in determining community structure alongside geographic positioning when classifying biogeographic groups in analyses.

## Conclusions

Our assessment of sharks and predatory fish assemblages across MPAs in the TEP identifies important considerations for protected area management in the region. Firstly, oceanic island MPAs support some of the largest shark abundances at nearshore reefs reported globally, establishing these MPAs as crucial shark hotspots and refuges from industrial fishing. While scientific research and conservation efforts of sharks has been increasing in the TEP, shark populations remain vulnerable to overfishing and climate change [24,111,112]. Notably, a substantial portion of the global shark fin trade originates from Eastern Pacific waters due to inadequate fisheries regulations and significant illegal, unregulated and unreported (IUU) fishing activity [18,19]. This largely unquantified exploitation continues to impact declining populations of shark species listed in the IUCN red list, such as the critically endangered *S. lewini* [59]. Well-designed fisheries management regulations in and around MPAs and coordinated management strategies across jurisdictions considering ecological spatial connectivity are therefore crucial to effectively conserve island shark populations which include migratory species [84,86,113–115].

Secondly, our findings reinforce previous evidence of depleted fish populations in Ecuadorian coastal MPAs and suggest potential fishing impacts in remote island MPAs. Given the slow recovery of long-lived predatory fishes depleted by fishing and the impending impacts of climate change [116,117], we emphasize the critical need for assessments of fishing impact in these MPAs as well as strengthened protection and enforcement. Cost-effective technologies, such as Automatic Identification System (AIS), alongside patrols could complement the latter and help prioritise effort across large or remote MPAs, or where they are infrequent. While assessments identifying signs of depletion in MPAs region-wide, presents a valuable opportunity to implement proactive rather than reactive management strategies. Our regional assessment of reef fish communities found evidence of fishing pressure effects among biogeographic patterns, highlighting the necessity to take measures to improve conservation-outcomes in both remote oceanic island and coastal MPAs throughout the TEP.

## Supporting information

S1 Table Multivariate composition analyses within biogeographic provinces and strong Canonical Analysis of Principal Coordinates (CAP) axis species correlations. Fixed-factor and pairwise PERMANOVA results testing differences in reef fish community composition among oceanic MPAs (S1A-B) and coastal MPAs (S1C-D), and reef fish species strong correlated (Pearson>0.7) with first and second CAP axes (S1E).
(XLSX)
